# Skeletal muscle desmin alterations following revascularization in peripheral artery disease claudicants

**DOI:** 10.1038/s41598-024-63626-3

**Published:** 2024-06-01

**Authors:** Dylan Wilburn, Dimitrios Miserlis, Emma Fletcher, Evlampia Papoutsi, Ahmed Ismaeel, Cassandra Bradley, Andrew Ring, Trevor Wilkinson, Robert S. Smith, Lucas Ferrer, Gleb Haynatzki, Peter Monteleone, Subhash Banerjee, Elizabeth Brisbois, William T. Bohannon, Panagiotis Koutakis

**Affiliations:** 1 Department of Biology, Baylor University, B.207 Baylor Science Building, One Bear Place #97388, Waco, TX 76798-7388 USA; 2 Department of Surgery and Perioperative Care, University of Texas, Austin, TX USA; 3 Department of Surgery, University of Texas Health Science Center San Antonio, San Antonio, TX USA; 4 Department of Physiology, University of Kentucky, Lexington, KY USA; 5 Department of Surgery, Baylor Scott & White Medical Center, Temple, TX USA; 6 Department of Biostatistics, University of Nebraska Medical Center, Omaha, NE USA; 7 Department of Internal Medicine, University of Texas, Austin, TX USA; 8 Department of Cardiology, Baylor Scott & White Medical Center, Dallas, TX USA; 9 School of Chemical, Materials and Biomedical Engineering, College of Engineering, University of Georgia, Athens, GA USA

**Keywords:** Physiology, Cardiovascular biology, Cardiovascular diseases, Vascular diseases, Peripheral vascular disease

## Abstract

Peripheral artery disease (PAD) is characterized by varying severity of arterial stenosis, exercise induced claudication, malperfused tissue precluding normal healing and skeletal muscle dysfunction. Revascularization interventions improve circulation, but post-reperfusion changes within the skeletal muscle are not well characterized. This study investigates if revascularization enhanced hemodynamics increases walking performance with concurrent improvement of mitochondrial function and reverses abnormal skeletal muscle morphological features that develop with PAD. Fifty-eight patients completed walking performance testing and muscle biopsy before and 6 months after revascularization procedures. Muscle fiber morphology, desmin structure, and mitochondria respiration assessments before and after the revascularization were evaluated. Revascularization improved limb hemodynamics, walking function, and muscle morphology. Qualitatively not all participants recovered normal structural architecture of desmin in the myopathic myofibers after revascularization. Heterogenous responses in the recovery of desmin structure following revascularization may be caused by other underlying factors not reversed with hemodynamic improvements. Revascularization interventions clinically improve patient walking ability and can reverse the multiple subcellular functional and structural abnormalities in muscle cells. Further study is needed to characterize desmin structural remodeling with improvements in skeletal muscle morphology and function.

## Introduction

Peripheral artery disease (PAD) is associated with the obstruction of peripheral arteries impairing blood flow to limbs. The most common clinical manifestation of symptomatic PAD is intermittent claudication (IC), which is defined as exercise induced pain caused by ischemia that is relieved with rest^1,2^. The alleviation of this pain during the cessation of exercise is due to decreased metabolic demands from the skeletal muscle and reperfusion of the tissue^3^. It has been postulated that exposure to repeated cycles of ischemia and reperfusion (I/R) can lead to a myopathy that progresses to further impair muscle function independent of hemodynamic obstruction^4–9^. PAD has been shown to cause increased ROS production from the mitochondria (electron transport complexes I, III, and IV) potentially indicating damaged and dysfunctional mitochondria^10–13^. Further, mitochondrial reactive oxygen species (ROS) production is normally neutralized by antioxidant defense systems, but concentrations of manganese superoxide dismutase are decreased with PAD indicating that this ROS neutralizing system may be compromised^11,14^. Uncontrolled ROS production can lead to a myriad of problems within the myofiber such as impaired anabolic signaling, increased protein catabolism, lipid peroxidation, and loss of mitochondria through mitophagy^15–17^.

Skeletal and cardiac muscle contains desmin, a major type III intermediate filament protein which constitutes one of many cytoskeletal structures within myofibers. Desmin is positioned near the sarcolemma at costameres, through the intermyofibrillar spaces, and around Z-discs of myofibrils in a honeycomb shape when viewed in anatomical cross-sections^18–21^. The structural positioning of desmin in both cardiac and skeletal muscle has been speculated to allows the cytoskeletal protein to have a major role in cellular homeostasis. In studies utilizing models of desminopathies, such as desmin null mice (Des^-/-^) or α-β-crystallin (CryAB) mutations, there is a marked decrease in mitochondrial function as well as altered mitochondrial localization^20,22^. Additionally, these models of desminopathies stimulate the localized loss and aggregation of desmin throughout the myofiber^20,22^. Similarly, Des^-/-^ mice display decreased sarcomere alignment and abnormal sarcomeres with no clear I or A bands, decreased muscle force, and muscular endurance^23^. Besides its structural role, desmin influences metabolic and contractile function within skeletal muscle and its turnover is likely highly regulated^20,22,23^.

Interestingly, investigations into PAD patients muscle morphology and function have shown muscle and myofiber atrophy as well as similar features identified in desminopathies^12,24,25^. PAD patients display both a sporadic pattern of desmin aggregates and a localized loss of desmin within myofibers^12,24^. Further, PAD IC patients display muscle atrophy, decreased muscular endurance, decreased muscular strength, as well as decreased mitochondrial function in gastrocnemius myofibers^12,24,25^. The point at which PAD skeletal muscle begins to worsen is unclear but involves alterations in the structure of desmin. The restoration of blood flow to the limb after revascularization has the potential to alter the trajectory of the muscle myopathy and may decrease the accumulation of desmin within the myofiber which may alleviate some negative symptoms of PAD. To the best of the authors knowledge no study has attempted to measure the morphological changes in the intermediate filament desmin, mitochondrial function, or walking performance within PAD skeletal muscle before and after revascularization interventions to determine if there were improvement in both morphology and metabolic function of the impacted skeletal muscle. Therefore, the aim of this paper is to determine if revascularization improves hemodynamics, skeletal muscle function, and improve the desminopathy that has been previously shown to exist in PAD patients.

## Methods

Fifty-eight patients referred for evaluation and treatment for lower extremity claudication from PAD per standard of care were recruited to participate in this study. The demographics of all patients are listed in Table 1. Each patient was assessed at the Baylor Scott and White Medical Center (Temple, TX) and the University of Texas Health Science Center San Antonio (San Antonio, TX), and the appropriate treatment was determined, resulting in a revascularization operation. Patients’ medical histories, ankle brachial index, 6-min walking ability, AND maximal walking performance were recorded. The initial muscle biopsy was collected during the revascularization procedure from the anterior medial side of the gastrocnemius. All patients returned to the corresponding Medical Center for their follow-up visit 6 months post-revascularization. For each patient, the same parameters were collected. The study was approved by Baylor University Institutional Review Board (human clinical trial NCT04089943 and single-IRB protocol 162404). Written informed consent was obtained from each patient who elected to participate. All methods were carried out in accordance with relevant guidelines and regulations.

**Table 1:** Patient Demographics at enrollment.

Age, years	62.2 ± 5.9
Body mass index	27.5 ± 6.3
Male/female	45/13
Race (W/WH/B)	36/18/4
Risk factors (%)	
Tobacco use	
Current	30 (51.7)
Never	17 (29.3)
Former	11 (19.0)
Hypertension	33 (56.9)
Diabetes mellitus	31 (53.4)
Dyslipidemia	46 (79.3)
CAD	16 (27.6)
Obesity, BMI > 30	14 (24.1)
Medications (%)	
Aspirin	22 (37.9)
ACE-inhibitor	27 (46.5)
ARB	6 (10.3)
β-Blocker	16 (27.6)
Calcium channel blocker	10 (17.2)
Surgery location (%)	
Aortoiliac	19 (32.8)
Femoropopliteal	17 (29.3)
Aortoiliac and femoropopliteal	22 (37.9)

*W* White, *WH* White Hispanic, *B* Black; *CAD* coronary artery disease.

### Hemodynamic measurements

Ankle brachial index (ABI) was measured at rest using Doppler technique (ABi Parks Flo Lab 2100-sx, Parks Medical Electronics, Inc., Aloha, OR) in the brachial artery and both posterior tibial and dorsalis pedis arteries^26^. ABI measurements were taken from the revascularized leg before and after revascularization.

### Walking performance testing

Walking performance tests were conducted one week before and six-months after revascularization operations. Patients were instructed to walk a 30 m distance as many times as possible within 6 min, and the distance achieved was recorded in meters. Additionally, maximal walking performance was measured for all PAD patients using the graded treadmill test. Briefly, patients walked at a constant speed of 3.2 km/h on a 0° grade that increased 2° every 2 min. Claudication onset time, claudication total distance, maximum walking time, and maximum walking distance were all measured during each test.

### Histology and quantitative fluorescence microscopy

Muscle samples collected for histology one week before and six-months after revascularization were immediately fixed in cold methacarn^12,13,24^ then transferred to cold ethanol:H_2_0 (50:50) after 48 h. Samples were then dehydrated in increasing gradients of ethanol before being cleared in xylene and paraffin embedded. Paraffin embedded tissues were subsequently sectioned at 4 μm, deparaffinized, and rehydrated in ethanol and distilled H_2_0 washes. The rehydrated slides were heated at 95 °C in 100 mM citrate buffer (pH 6.0) for 15 min and cooled for 20 min before being soaked in washing buffer for 30 min at room temperature. Slides were then incubated with a desmin primary rabbit monoclonal antibody (0.20 μg/ml; ab32362, Abcam) overnight at 4 °C. The next day, slides were incubated with the secondary antibody Fluor® 488 goat anti-rabbit IgG (H + L) antibody, highly cross-adsorbed (10 μg/mL; A-11034; Life Technologies, Molecular Probes). Sarcolemmas were labeled with Alexa Fluor® 647-conjugated wheat germ agglutinin (10 μg/ml; W32466; Life Technologies, Molecular Probes) and slides were mounted in ProLong® Gold anti-fade medium with DAPI nuclear stain (P36931; Life Technologies, Molecular Probes).

Desmin quantification was based on three-channel imaging of each microscopy field as previously described^12,27–29^. Briefly, fluorescence images were captured at 10 × objective of a wide field epifluorescences microscope and a PRIME 95B sCMOS camera. All tissue that was mounted on each slide (250–350 microscopic fields per specimen, corresponding to 20,000 to 30,000 myofibers and in triplicate) was captured in three fluorescence channels corresponding to nuclei, desmin, and myofiber sarcolemma. Fluorescence signal produced by desmin was expressed as mean pixel intensity in grayscale units. The florescence was corrected for background intensity near the black level of the camera, negative stained control slides and all myofibers mean desmin intensity for each sample was determined. Myofiber cross-sectional area (CSA), perimeter, roundness, solidity, major axis length, minor axis length, and myofiber density were all measured for each participant.

### Mitochondrial respiration

Muscle samples partitioned for mitochondrial respiration were immediately placed into an ice-cold preservation solution and immediately transferred to the lab for processing. Muscle samples were saponin-permeabilized before respiration testing with an Oroborors O2k Oxygraph (Oroboros Instruments, Innsbruck, Austria) as previously conducted^30^. Briefly, saponin permeabilized samples were immersed in respiration media consisting of 105 mM MES potassium salt, 30 mM potassium chloride (KCl), 10 mM potassium dihydrogen phosphate (KH_2_PO4), 5 mM magnesium chloride, hexahydrate (MgCl_2_.6H2O), and 0.5 mg/mL BSA. Oxygen consumption was measured at 37 °C in a respiration buffer supplemented with creatine monohydrate (20 mM). A substrate inhibitor titration protocol was performed, whereby 2 mM malate and 10 mM glutamate were added to the chambers followed by the addition of 4 mM ADP, to measure Complex I respiration. Next, 10 mM succinate was added to the chambers to stimulate electron flow through Complex II. Rotenone (10 μM) was then used to inhibit Complex I, and 1 mM of duroquinol was added to measure Complex III. Finally, we added 5 μM antimycin A to inhibit Complex III. followed by 0.4 mM N,N,N’,N’-tetramethyl-p-phenylenediamine (TMPD), and 2 mM ascorbate to prevent TMPD auto-oxidation to measure Complex IV. All reagents and chemicals were purchased from Sigma Aldrich (St. Louis, MO). Citrate Synthase (CS) was measured using a modified protocol from Janssen and Boyle^31^ in skeletal muscle lysates in duplicate as a maker of mitochondrial content. All oxygen consumption values for each individual mitochondrial complex were made relative to CS activity for each participant. The activity of Mn SOD (Superoxide Dismutase) activity was measured using a previously published method^14^ and normalized to CS activity.

### Statistical analysis

Multivariate analyses of variance using general linear models with repeated measurements of pre-post revascularization operations were performed. These models were selected to consider the multiple dependent variables measured from each patient. Statistical power analyses established that this sample size had sufficient power (0.97) to detect a small to medium effect size correlation, r = 0.20 with α = 0.05 (G*Power 3.1.9.). GraphPad Prism was used to create the figures. All statistical analyses were conducted using the commercially available statistical software SPSS version 28.0 (IBM, Armonk, NY, USA).

## Results

### Hemodynamics

The revascularization surgery significantly improved the average measures of hemodynamic blood flow within the patients (Table 2). ABI increased substantially 6 months post-surgery (*p* < 0.001).

**Table 2:** Ankle Brachial Index, desmin and myofiber morphometrics of the gastrocnemius of PAD patients at baseline and 6 months after revascularization.

	Pre- revascularization	Post- revascularization	*p*-value
Ankle Brachial Index	0.49 ± 0.24	0.77 ± 0.29	< 0.001
Desmin (gsu)	1213 ± 376	1072 ± 274	0.034
Cross-sectional area (μm^2^)	4206 ± 1450	4725 ± 1659	0.022
Major axis (μm)	98.6 ± 19.5	104 ± 19.6	0.033
Minor axis (μm)	50.7 ± 9.6	54.4 ± 10.9	0.045
Equivalent diameter (μm)	70.1 ± 12.7	74.4 ± 13.4	0.024
Perimeter (μm)	270 ± 50.4	286 ± 53.4	0.058
Roundness	0.820 ± 0.03	0.817 ± 0.02	0.880
Solidity	0.914 ± 0.03	0.917 ± 0.02	0.270
Myofiber density	0.716 ± 0.08	0.764 ± 0.06	0.001

Revascularization operation improved the Ankle Brachial Index, desmin and myofiber morphometrics, indicating that it is an effective treatment option for patients with PAD.

### Myofiber morphometrics

Revascularization yielded several beneficial changes in the myofiber morphology 6 months following the operation and can be seen in Table 2. There were increases seen in the average myofiber cross sectional area (*p* = 0.022), major axis (*p* = 0.033), minor axis (*p* = 0.045), equivalent diameter (*p* = 0.024), and myofiber density (*p* = 0.001), There was no statistically significant difference in the other parameters.

**Figure 1:**
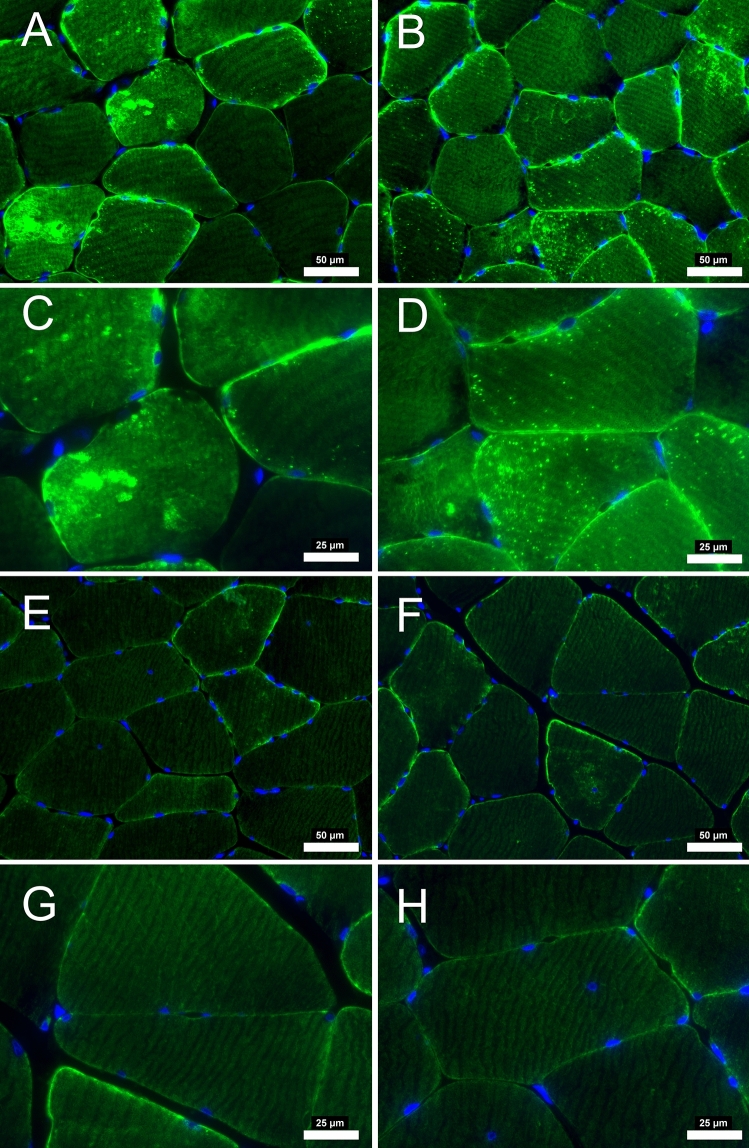
Desmin immunofluorescence staining from patients with PAD that improved after revascularization operation. At pre-revascularization (**A**-**B**, 20 × objective) disorganization of desmin is evident with areas of higher concentration within the myofiber (**C**-**D**, 40 × objective). After post-revascularization (**E**–**F**, 20 × objective) desmin concentration within the myofiber is decreased. Subsarcolemmal desmin is mainly evident (**G-H**, 40 × objective) indicating a relative normal staining. Myofiber morphology is improved with myofibers having a similar shape and size after revascularization operations. Scale bars are 20 × objective at 50 μm and 40 × objective at 25 μm. Desmin is stained with green and nuclei with blue.

**Figure 2:**
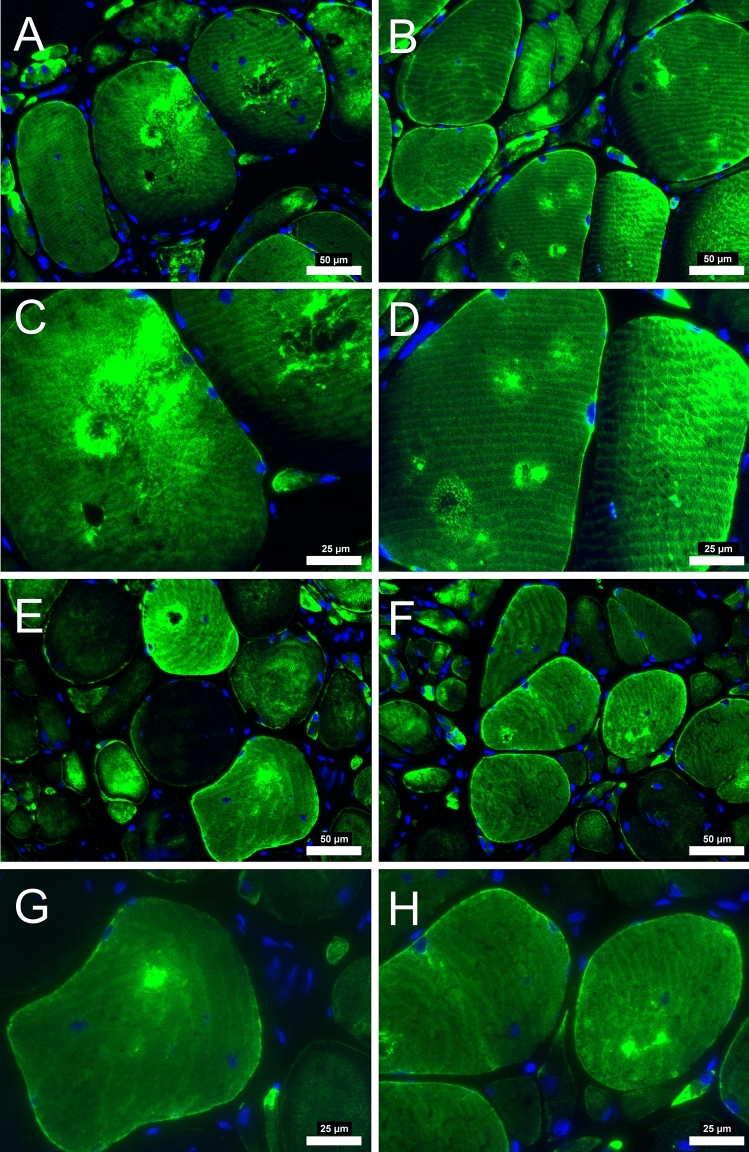
Desmin immunofluorescence staining from patients with PAD that did not improve after revascularization operation. At pre-revascularization (**A**-**B**, 20 × objective), the disorganization of desmin is evident in several areas of higher concentration within the myofiber (**C**-**D**, 40 × objective). After post-revascularization (**E**–**F**, 20 × objective), desmin concentration within the myofiber did not decrease. Myofiber morphology did not improve, with myofibers having irregular shape and size after revascularization operations. Scale bars are 20 × objective at 50 μm and 40 × objective at 25 μm. Desmin is stained green, and the nuclei are blue.

There was a significant decrease in total amount of average desmin quantified from the fluorescence microscopy analysis (*p* = 0.034). In addition to the average decrease, there was a clear difference in the appearance of the desmin fluorescence within each patient after revascularization. There appear to be focal losses of the desmin protein that are absent of signal, while other locations consist of desmin aggregates that fluoresce intensely in all participants before surgery. After revascularization, the myofibers from some patients (n = 50) appeared to have decreased number of desmin aggregates and refilled the locations where desmin was previously absent (Fig. 1). However, some patients (n = 8) appear to have been less affected or had no change in the desmin structure from the revascularization (Fig. 2). For reasons currently unknown, the focal absence of desmin and aggregates of desmin persisted despite the improvement in hemodynamics within these individuals. These results indicate that a group of PAD patients will respond and reverse the pathological desmin morphology while others respond differently to revascularization treatments.

### Walking performance

**Figure 3:**
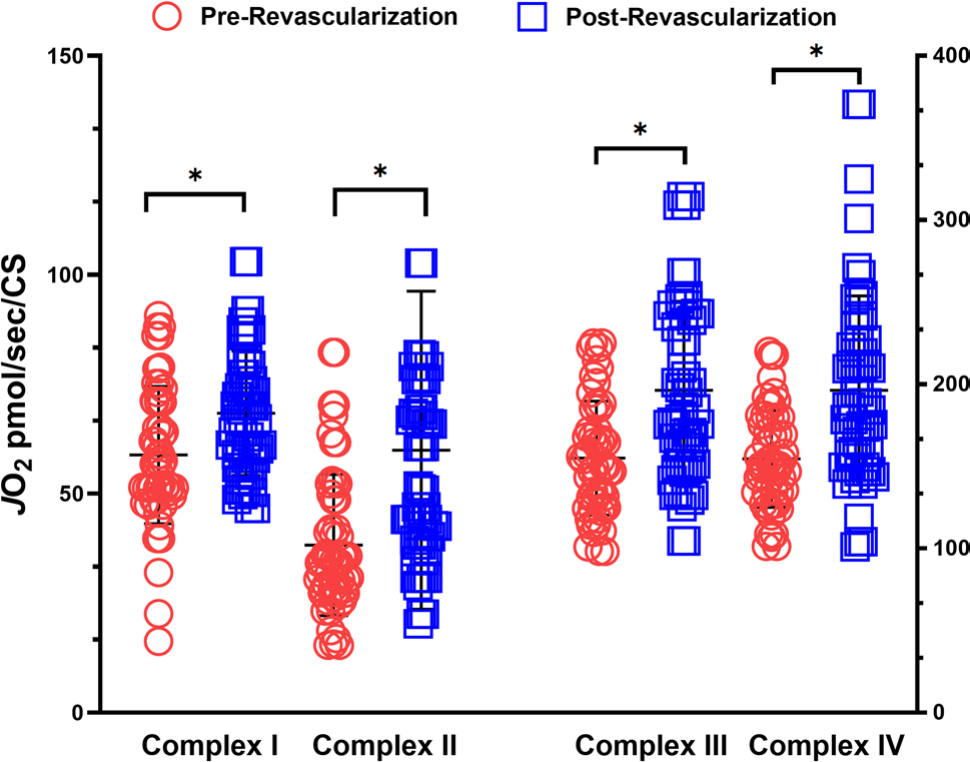
Revascularization operations improved mitochondrial respiration of the electron transport chain complexes I-IV. Mitochondrial respiration is normalized to citrate synthase activity. *p < 0.05.

There were substantial improvements in the average walking performance after the revascularization procedure that are presented in Fig. 3. 6 min walking distance (SMWD) improved significantly from their pre-revascularization (277 m) values to post-revascularization (365 m, *p* < 0.001). Similarly, measures from the graded treadmill test also improved after revascularization with increases in claudication onset time (COT; 112 vs. 170 s, *p* = 0.001), claudication onset distance (92 vs. 189 m, *p* = 0.001), maximal walking time (MWT; 254 vs. 628 s, *p* = 0.001), and maximum walking distance (MWD; 235 vs. 617 m, *p* < 0.001).

### Mitochondrial respiration and antioxidant activity

**Figure 4:**
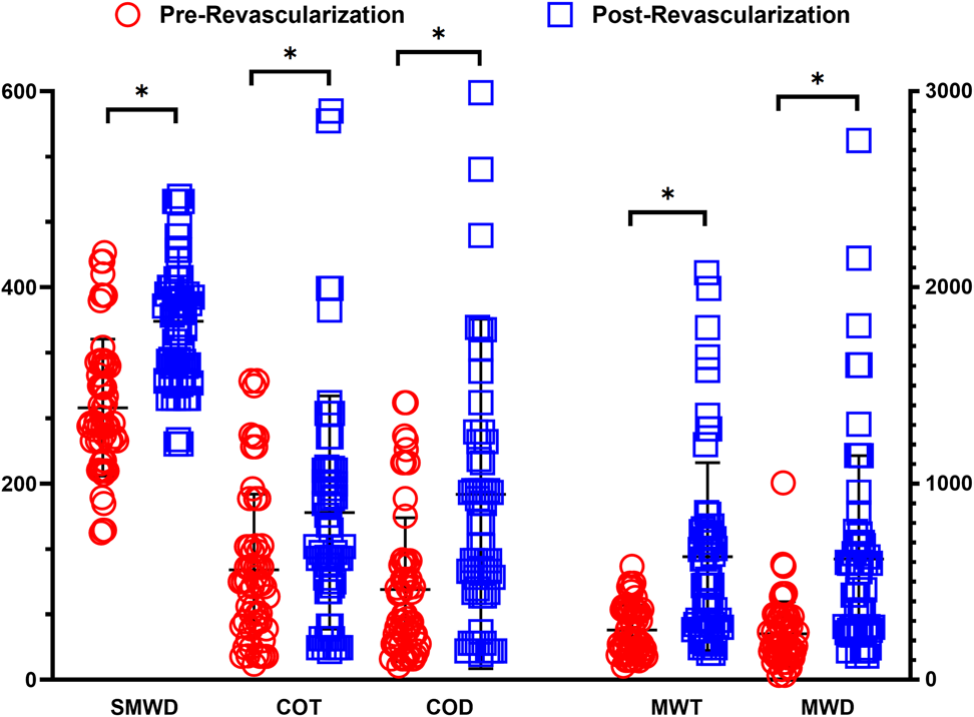
Revascularization operations improved the walking function of patients with PAD. Six minute walking distance (SMWD), claudication onset time (COT), claudication onset distance (COD), maximum walking time (MWT), and maximum walking distance (MWD) were all significantly improved post-revascularization. *p < 0.05.

The revascularization procedure led to improvements in oxygen consumption after revascularization operations (Fig. 4). There were statistically significant increases in the total oxygen consumed for complex I (*p* = 0.037), complex II (*p* = 0.012), complex III (*p* = 0.040), and complex IV (*p* = 0.044). Total SOD activity normalized by CS activity was not statistically different (0.241 vs. 0.194 units per CS).

## Discussion

Improved understanding of what happens during the clinical course and clinical care of ischemic tissue in PAD could hold the potential to improve our clinical identification of tissue needing reperfusion, our interventional therapies as well as our ability to procedurally target which patients will improve with revascularization. Furthermore, pharmaceutical therapies and noninvasive treatments can all likely be advanced by an enhanced understanding of the metabolic changes within ischemic tissue. Previous work demonstrates that revascularization improves hemodynamic measures associated with PAD progression and improves cardiovascular disease risk^32–35^. Improvement in ABI and an increase in average walking time may also decrease the frequency of I/R cycles and delay the progression of myopathy within these patients. However, the functional improvements in walking ability with revascularization are not always seen^35–37^. One study found that revascularization did not reverse the clinical functional decline in muscle performance in some patients^37^. Walking speed, step cadence, and 6-min walking ability were identical 3–4 months post-surgery^37^. The persistent impairments seen within these individuals after revascularization may potentially be due to differences in disease stage or a varying presentation of PAD that may be due to the limited ability of our current guidelines and clinical tests to stratify^36,37^.

Improvements in walking performance were associated with increased perfusion of the affected limb and were accompanied by substantial improvements in mitochondrial respiration across all electron transport chain complexes 6 months post-revascularization. Revascularization also led to a decrease in the activity of total SOD. Previous research has shown basal decreases in the activity of the SOD antioxidant enzyme within PAD patients that did not receive revascularization^14^. It is difficult to determine if reduced SOD is found across all stages of PAD since the study did not stratify the PAD based on staging^14^. In the context of the current study, the decrease in SOD activity could possibly be related to a reduced ROS load experienced after revascularization. Typically, ROS production has been shown to occur at complex I, III, and IV^10–13^. Collectively, the improvements in JO_2_ (mitochondrial respiratory oxygen flux) associated with these complexes, improvements in walking performance, and increased perfusion likely indicate improvements in individual electron transport chain complex function and reduced ROS production. Further, research investigating the alterations of antioxidant defense enzymes content and activity are needed to clearly understand these changes within this demographic.

The average changes in the CSA and morphological characteristics of myofibers after the surgical intervention indicate that revascularization has the potential to reverse the muscle myopathy within IC PAD patients. Further, the quantified amount of desmin present within each of the participants showed an average decrease after revascularization. However, upon investigating morphological changes in desmin structure, there were still desmin aggregates and complete loss of desmin in various locations within the myofibers of some patients after revascularization. The presence of desmin aggregates in the patients either before and after surgery may be formed by mutations, post-translational modifications or oxidation mediated changes that decreases CryAB’s chaperone function^38–40^. Desmin has previously been found to be a major target for oxidation within skeletal muscle^41^. If desmin is being oxidized during I/R cycles, it could form protein aggregates via ROS induced unfolding. However, there is limited evidence on the effect of desmin aggregation within PAD skeletal muscle, and it cannot be ruled out that both CryAB and desmin may mutually contribute to abnormal cellular structure and clinical presentation. However, the persistence of desmin aggregation in patients who displayed improved hemodynamics, mitochondrial function, and decreased Mn-SOD activity after surgery may indicate that ROS-mediated changes in desmin structure are not the only potential mechanism for desmin aggregate formation. The time frame and natural progression of this hypothetical mechanism for continued functional decline is still unclear.

The patients that displayed no change in desmin architecture after revascularization still showed focal loss of desmin in locations throughout the myofiber. This decrease in desmin content in specific locations may indicate spatially regulated increases in desmin catabolism not restored by revascularization. Desmin degradation was shown to be a target of calpain proteolysis and precedes sarcomere breakdown upon atrophying stimuli^42–44^. The initial step that facilitates desmin disassembly by calpain-1 is the phosphorylation of desmin by glycogen synthase kinase 3-β (GSK3-β)^42^. Inhibition of GSK3-β using L803mts has been shown to prevent desmin phosphorylation and myofibril destruction^42^. For desmin depolymerization and degradation to occur it seems necessary that calpain-1 becomes active to fulfill this action. It has been postulated that calpains have the potential to become active after exercise-induced calcium release (with or without damage) and can have elevated activity for 95 h after intense exercise^45–47^. Exercising in a hypoxic environment was shown to cause higher neuromuscular activation for each muscle within the quadriceps muscle group^48^. The hypoxic intramuscular environments during frequent I/R cycles could cause compensatory increases in both calcium concentration and metabolic stress facilitating GSK3-β/calpain induced desmin loss. The specific mechanism for region specific focal loss of desmin is also unclear and further research is necessary to shed light in a potentially underlying mechanism for patient presentation.

It may be possible that specific intermyofibrillar regions may be undergoing different remodeling/signaling processes in response to metabolic stress at the same time. For example, distinct pockets of mitochondria could be producing large amounts of ROS which may explain regions with desmin aggregates, while regionally specific GSK3-β/calpain activation could cause the absence of desmin. It is perplexing why revascularization would lead to the recovery of desmin structure in some patients but not others. Previous work with cardiomyocytes has shown irreversible damage occurs after I/R if the ischemic episode last for an extended duration of time^49,50^. Perhaps a similar mechanism, albeit much slower, occurs in PAD whereby the total exposure or frequency of exposure to I/R cycles and the severity of the I/R is a predictive indicator of myopathic progression. Further research is needed to elucidate these speculative mechanisms that underpin the observed remodeling and potentially explain the dichotomous responsiveness to revascularization.

## Limitations

While the results of this study are promising there are limitations that should be considered. The number of patients and time points of data collection are limited. It is also possible that the individuals we consider to be non-responders may recover over a longer period of time with more medical supervision. Further, without direct supervision of the patients, lifestyle behaviors could also contribute to delayed skeletal muscle desmin remodeling. Future studies will have closer and more prolonged annual follow up.

## Conclusion

Studies that investigate changes in clinical walking ability and revascularization in PAD patients show inconsistent positive outcomes. Further research is needed to shed light on the underlying mechanisms of treatment and potentially integrate novel structural and biochemical tests to provide a prognostic value for clinical decision making. It may be that in certain patients with IC, the skeletal muscle, despite improved perfusion, display subcellular functional and architectural impairments, which could explain different responses to the standard of care treatments and disease progression. We demonstrate that the intermediate cytoskeletal protein desmin remains perturbed after revascularization in certain patients with IC. Considering these findings, further research in the underlying mechanisms could lead to markers with prognostic value in the claudication group of patients who are considered for interventional therapies.

## Data Availability

Study data are available from the corresponding author upon reasonable request.

## References

[1] Rutherford RB, Baker JD, Ernst C, Johnston KW, Porter JM, Ahn S (1997). Recommended standards for reports dealing with lower extremity ischemia: Revised version. J. Vasc. Surg..

[2] Gornik HL, Beckman JA (2005). Cardiology patient page Peripheral arterial disease. Circulation.

[3] Norgren L, Hiatt WR, Dormandy JA, Nehler MR, Harris KA, Fowkes FG (2007). Inter-society consensus for the management of peripheral arterial disease (TASC II). J. Vasc. Surg..

[4] Pipinos II, Juadge AR, Selsby JT, Zhu Z, Swanson SA, Nella AA (2008). The myopathy of peripheral arterial occlusive disease: Part 1. Functional and histomorphological changes and evidence for mitochondrial dysfunction. Vasc. Endovasc. Surg..

[5] Pipinos II, Judge AR, Selsby JT, Zhu Z, Swanson SA, Nella AA (2008). The myopathy of peripheral arterial occlusive disease: Part 2. Oxidative stress, neuropathy, and shift in muscle fiber type. Vasc. Endovasc. Surg..

[6] Steven S, Daiber A, Dopheide JF, Munzel T, Espinola-Klein C (2017). Peripheral artery disease, redox signaling, oxidative stress - Basic and clinical aspects. Redox Biol..

[7] McDermott MM, Ferrucci L, Gonzalez-Freire M, Kosmac K, Leeuwenburgh C, Peterson CA (2020). Skeletal muscle pathology in peripheral artery disease: A brief review. Arterioscler. Thromb. Vasc. Biol..

[8] Liang Z, Zhang W, Zhu T, Li Y, Cao P, Wu Y (2021). Ischemia-reperfusion injury in peripheral artery disease and traditional Chinese medicine treatment. Evid. Based Complement Alternat. Med..

[9] Eltzschig HK, Collard CD (2004). Vascular ischaemia and reperfusion injury. Br. Med. Bull..

[10] Makris KI, Nella AA, Zhu Z, Swanson SA, Casale GP, Gutti TL (2007). Mitochondriopathy of peripheral arterial disease. Vascular.

[11] Koutakis P, Ismaeel A, Farmer P, Purcell S, Smith RS, Eidson JL (2018). Oxidative stress and antioxidant treatment in patients with peripheral artery disease. Physiol. Rep..

[12] Koutakis P, Miserlis D, Myers SA, Kim JK, Zhu Z, Papoutsi E (2015). Abnormal accumulation of desmin in gastrocnemius myofibers of patients with peripheral artery disease: Associations with altered myofiber morphology and density, mitochondrial dysfunction and impaired limb function. J. Histochem. Cytochem. Off. J. Histochem. Soc..

[13] Koutakis P, Weiss DJ, Miserlis D, Shostrom VK, Papoutsi E, Ha DM (2014). Oxidative damage in the gastrocnemius of patients with peripheral artery disease is myofiber type selective. Redox Biol..

[14] Pipinos II, Judge AR, Zhu Z, Selsby JT, Swanson SA, Johanning JM (2006). Mitochondrial defects and oxidative damage in patients with peripheral arterial disease. Free Radic. Biol. Med..

[15] Ismaeel A, Brumberg RS, Kirk JS, Papoutsi E, Farmer PJ, Bohannon WT (2018). Oxidative stress and arterial dysfunction in peripheral artery disease. Antioxidants.

[16] Ismaeel A, Lavado R, Smith RS, Eidson JL, Sawicki I, Kirk JS (2018). Effects of limb revascularization procedures on oxidative stress. J. Surg. Res..

[17] Thompson JR, Swanson SA, Haynatzki G, Koutakis P, Johanning JM, Reppert PR (2015). Protein concentration and mitochondrial content in the gastrocnemius predicts mortality rates in patients with peripheral arterial disease. Ann. Surg..

[18] Lazarides E (1980). Intermediate filaments as mechanical integrators of cellular space. Nature.

[19] Desmin cytoskeleton: A potential regulator of muscle mitochondrial behavior and function, (2002). 10.1016/s1050-1738(02)00184-6 12536120

[20] Capetanaki Y, Bloch RJ, Kouloumenta A, Mavroidis M, Psarras S (2007). Muscle intermediate filaments and their links to membranes and membranous organelles. Exp. Cell Res..

[21] Capetanaki Y, Milner DJ, Weitzer G (1997). Desmin in muscle formation and maintenance: Knockouts and consequences. Cell Struct. Funct..

[22] Maloyan A, Sanbe A, Osinska H, Westfall M, Robinson D, Imahashi K (2005). Mitochondrial dysfunction and apoptosis underlie the pathogenic process in alpha-B-crystallin desmin-related cardiomyopathy. Circulation.

[23] Li ZL, Mericskay M, Agbulut O, ButlerBrowne G, Carlsson L, Thornell LE (1997). Desmin is essential for the tensile strength and integrity of myofibrils but not for myogenic commitment, differentiation, and fusion of skeletal muscle. J. Cell Biol..

[24] Koutakis P, Myers SA, Cluff K, Ha DM, Haynatzki G, McComb RD (2015). Abnormal myofiber morphology and limb dysfunction in claudication. J. Surg. Res..

[25] McDermott MM, Guralnik JM, Albay M, Bandinelli S, Miniati B, Ferrucci L (2004). Impairments of muscles and nerves associated with peripheral arterial disease and their relationship with lower extremity functioning: the InCHIANTI Study. J. Am. Geriatr. Soc..

[26] Farah BQ, Ritti-Dias RM, Montgomery PS, Casanegra AI, Silva-Palacios F, Gardner AW (2016). Sedentary behavior is associated with impaired biomarkers in claudicants. J. Vasc. Surg..

[27] Weiss DJ, Casale GP, Koutakis P, Nella AA, Swanson SA, Zhu Z (2013). Oxidative damage and myofiber degeneration in the gastrocnemius of patients with peripheral arterial disease. J. Transl. Med..

[28] Huang D, Casale GP, Tian J, Wehbi NK, Abrahams NA, Kaleem Z (2007). Quantitative fluorescence imaging analysis for cancer biomarker discovery: Application to beta-catenin in archived prostate specimens. Cancer Epidemiol. Biomark. Prev. Publ. Am. Assoc. Cancer Res. Cosponsored Am. Soc. Prev. Oncol..

[29] Cluff K, Miserlis D, Naganathan GK, Pipinos II, Koutakis P, Samal A (2013). Morphometric analysis of gastrocnemius muscle biopsies from patients with peripheral arterial disease: objective grading of muscle degeneration. Am. J. Physiol. Regul. Integr. Comparat. Physiol..

[30] Ismaeel A, Fletcher E, Miserlis D, Wechsler M, Papoutsi E, Haynatzki G (2022). Skeletal muscle MiR-210 expression is associated with mitochondrial function in peripheral artery disease patients. Transl. Res..

[31] Janssen RC, Boyle KE (2019). Microplate assays for spectrophotometric measurement of mitochondrial enzyme activity. Methods Mol. Biol..

[32] Watanabe K, Takahashi H, Watanabe T, Otaki Y, Kato S, Tamura H (2020). Endovascular revascularization improves the central hemodynamics and augmentation index in patients with peripheral artery disease. Intern. Med..

[33] Giugliano G, Perrino C, Schiano V, Brevetti L, Sannino A, Schiattarella GG (2012). Endovascular treatment of lower extremity arteries is associated with an improved outcome in diabetic patients affected by intermittent claudication. BMC Surg..

[34] Miller AJ, Luck JC, Kim DJ, Leuenberger UA, Aziz F, Radtka JF (2018). Peripheral revascularization attenuates the exercise pressor reflex and increases coronary exercise hyperemia in peripheral arterial disease. J. Appl. Physiol..

[35] Regensteiner JG, Hargarten ME, Rutherford RB, Hiatt WR (1993). Functional benefits of peripheral vascular bypass surgery for patients with intermittent claudication. Angiology.

[36] Hogan SE, Nehler MR, Anand S, Patel MR, Debus S, Jackson MT (2022). Improvement in walking impairment following surgical and endovascular revascularization: Insights from VOYAGER PAD. Vasc. Med..

[37] Gardner AW, Killewich LA (2001). Lack of functional benefits following infrainguinal bypass in peripheral arterial occlusive disease patients. Vasc. Med..

[38] Hoover HE, Thuerauf DJ, Martindale JJ, Glembotski CC (2000). alpha B-crystallin gene induction and phosphorylation by MKK6-activated p38 - A potential role for alpha B-crystallin as a target of the p38 branch of the cardiac stress response. J. Biol. Chem..

[39] Nagaraj RH, Nahomi RB, Mueller NH, Raghavan CT, Ammar DA, Petrash JM (2016). Therapeutic potential of alpha-crystallin. Biochim. Biophys. Acta.

[40] Wang X, Osinska H, Klevitsky R, Gerdes AM, Nieman M, Lorenz J (2001). Expression of R120G-alphaB-crystallin causes aberrant desmin and alphaB-crystallin aggregation and cardiomyopathy in mice. Circ. Res..

[41] Biol AJ, Odena MA, Oliveira E, Olive M, Ferrer I (2007). Desmin is oxidized and nitrated in affected muscles in myotilinopathies and desminopathies. J. Neuropathol. Exp. Neur..

[42] Aweida D, Rudesky I, Volodin A, Shimko E, Cohen S (2018). GSK3-beta promotes calpain-1-mediated desmin filament depolymerization and myofibril loss in atrophy. J. Cell Biol..

[43] Volodin A, Kosti I, Goldberg AL, Cohen S (2017). Myofibril breakdown during atrophy is a delayed response requiring the transcription factor PAX4 and desmin depolymerization. Proc. Natl. Acad. Sci. USA.

[44] Cohen S, Zhai B, Gygi SP, Goldberg AL (2012). Ubiquitylation by Trim32 causes coupled loss of desmin, Z-bands, and thin filaments in muscle atrophy. J. Cell Biol..

[45] Baylor SM, Hollingworth S (2003). Sarcoplasmic reticulum calcium release compared in slow-twitch and fast-twitch fibres of mouse muscle. J. Physiol..

[46] Armstrong RB, Warren GL, Warren JA (1991). Mechanisms of exercise-induced muscle fibre injury. Sports Med..

[47] Raastad T, Owe SG, Paulsen G, Enns D, Overgaard K, Crameri R (2010). Changes in calpain activity, muscle structure, and function after eccentric exercise. Med. Sci. Sports Exerc..

[48] Yoshiko A, Katayama K, Ishida K, Ando R, Koike T, Oshida Y (2020). Muscle deoxygenation and neuromuscular activation in synergistic muscles during intermittent exercise under hypoxic conditions. Sci. Rep..

[49] Williams RS, Benjamin IJ (2000). Protective responses in the ischemic myocardium. J. Clin. Invest..

[50] Jennings RB, Ganote CE (1974). Structural changes in myocardium during acute ischemia. Circ. Res..

